# Patterns of psychiatric admissions and predictors of patient’s outcome in Jimma University Teaching and Referral Hospital: a retrospective study

**DOI:** 10.1186/s13033-017-0148-0

**Published:** 2017-06-14

**Authors:** Sinan Tadesse, Abraham Tamirat Gizaw, Getachew Kirose Abraha, Lakew Abebe Gebretsadik

**Affiliations:** 1 Department of Internal Medicine, Public Health and Medical Sciences College, Jimma University, P. O. Box: 378, Jimma, Ethiopia; 2 Department of Health Education and Behavioural Sciences, Public Health and Medical Sciences College, Jimma University, Jimma, Ethiopia

**Keywords:** Psychiatric Patient Care Outcome, Psychiatry Recovery, JUTH

## Abstract

**Background:**

Psychiatric morbidity burden accounts 12.45% of the disease admission burden in Ethiopia; only two referral hospitals are found to manage all cases. The aim of this study is to assess the predictors of patient outcomes.

**Method:**

A 3 years retrospective patients’ cards, charts and medical notes review in psychiatry case admission department of Jimma university teaching and training specialised hospital was conducted. All the admitted cases included in this study. Bivariate and multivariable logistic regression analyses were conducted to identify independent predictors of outcomes.

**Result:**

Among 402 study participants, the majority of them 301 (74.9%), were improved from their mental illnesses. First to eight grades completed study participants were found to be 1.34 times more likely improved mental illness than not able to read or write [AOR = 1.34, 95% CI (1.18–2.78), P < 0.009)]. The probability of improving from mental illness on married study participants was found 2.81 times more likely than single study participants [AOR = 2.81, CI (1.90–4.50), P < 0.043]. First time admitted cases improved 2.82 times more likely than those having a previous admission history [AOR = 2.82, CI (2.05–3.17), P < 0.05]. Duration of stay from 31 to 44 days showed more likely than from 1 to 20 days on patient improvement, [AOR = 1.88, CI (1.42–2.65), P < 0.034]. However, the hospital stay above 44 days does not show any statistical association with patient’s medical improvement.

**Conclusion:**

Married, better educated, and the hospital stay of one to one-and-half month predicts better health outcome. Thus, this study suggests, psychiatric case management needs the collaborative care of the family in concurrence with counselling and guidance with enough time to better-off patients’ outcomes. Our findings are useful in designing and improving—patient services for psychiatric patient programs and focused health communication and counselling strategies in relation to psychoactive substances in Ethiopia.

## Background

Mental health is an important component of health according to the World Health Organization (WHO) definition, which states health as a complete state of physical, mental and social wellbeing, not merely the absence of disease and infirmity. The term mental illness (psychiatric disorders) encompasses significant clinical syndromes with behavioural and psychological symptoms of distress or impairment in functioning [1]. It has been estimated that globally, half a billion people suffering from mental disorder or impairment [2]. Over 150 million people suffer from depression, 24 million people from schizophrenia, billions of abuse substances and 800,000 people die from suicide each year. Mental and behavioural disorders are estimated to account for 12% of the global burden of disease [3].

It is evident that mental health unswervingly related to the growing complexity of the low-income country’s society, a large proportion are being removed from the protection of the simple traditional environments to the complex, heterogeneous and less protective modern ones. Problems such as unemployment, economic and political instability, inflation, overcrowding, crime, divorce, broken homes, prostitution, drug addiction and lack of educational opportunities now abound. These are indices of social disorganisation, which have significant implications for mental health [4].

In near to the ground income country, where malnutrition and infectious diseases are common, mental disorders which are regarded as not life threatening are given low priority. As a result, the world is suffering from increasing burden of mental disorders [4, 5]. The provision of adequate, fair and equitable care for patients in the developing world, particularly in sub-Saharan Africa is one of the greatest challenges of the 21st-century health system [6].

The “unfinished agenda” of infectious diseases remains a legitimate priority, but the burden of psychiatric disorders and other chronic diseases continues to grow unchecked. These concerns are compounded by un-moderated population growth, continuing economic constraints and unparalleled socioeconomic and geopolitical changes. Psychiatric disorders constitute four of the top ten causes of worldwide disease burden [7], yet remain lowest on the agenda of policy-makers, particularly in developing countries [8].

Integrating psychiatric care into primary care, the principal strategy to address the mental health unmet need has remained inadequate and psychiatric services have been centralised in the large cities in sub-Saharan Africa. This is no different in Ethiopia, where the bulk of the care is provided by Amanuel Hospital, located in Addis Ababa. Despite the development of several regional psychiatric units, there is evidence for the limited use of services in the wider community. Which clearly shows within one of the largest ever community-based studies, conducted in southern-central Ethiopia, fewer than 10% of patients with schizophrenia and bipolar disorder had received psychiatric treatment [9].

In Ethiopia, mental illness is the leading non-communicable disorder in terms of burden, comprised 12.45% of the total burden of disease, and with schizophrenia and depression included in the top ten most burdensome conditions [2, 9].

Some of the predisposing factors are genetic, age, sex and race, while common precipitating factors include environmental factors, family, social, culture, economic, marital status, occupation, alcohol ingestion, physical defects and illnesses and social isolation [5, 10].

According to most Ethiopians’ culture, mental illnesses are generally believed to be caused by evil or supernatural force and physical causes are rarely considered for mental problems. As a result, very few patients with mental illnesses come to health institutions and most Ethiopian people use traditional medicine or ‘Holy water’ for a treatment of mental illnesses; and even those who come to health institutions after they have tried and failed the traditional treatment in most cases [11].

Currently, the care provided by Jimma University Teaching and Training Specialized Hospital of psychiatric facility serves as an index of the level of psychiatric care in the southwest of Ethiopia. Thus, this study was intended to assess patterns of psychiatric admission and the predictors of patient’s case management outcome that may also provide greater insight into research endeavours and psychiatric disorder prevention and case management programs interventions.

## Methods

### Study design and setting

A retrospective review of patient admissions to the psychiatric unit at Jimma University Teaching Hospital (JUTH) September 2012 to September 2015 was conducted. The data retrieved from standard Health Management Information System (HMIS) logbook. The hospital is located in Jimma City, 357 km away from Addis Ababa, in the southwest part of Ethiopia. It is a referral centre for the southwestern part of Ethiopia, and currently, the only teaching and referral hospital in the southwestern part of the country. It provides services for approximately 9000 inpatient and 80,000 outpatient attendances a year who are visiting the hospital from the catchment population. The psychiatric facility gives both outpatient and inpatient services for more than 30 million populations, which served a huge bulk of the community next to Paulos Hospital in Addis Ababa Ethiopia concerning Psychiatry health facility.

### Participants and data collection procedure

A total of 410 psychiatric admissions were recorded in the registration book for the period traversing September 2012 to September 2015. Out of 410 admissions reviewed, detailed documentation was available for 402 admissions. The psychiatry resident students under the supervision of a psychiatrist recorded the preliminary data in English. Data was charted on a structured paper format developed for the purpose of this study by the psychiatry residents and under the supervision of a clinical officer in mental health. The data was then coded and entered into the statistical software. Recorded data included, socio-demographic characteristics, place of residence, duration of admission (in days), whether the patient had to pay for the hospital care, diagnosis on discharge, and outcome at discharge (improved, absconded, no change and deceased). The Diagnostic Statistical Manual 4th revision (DSM-IV) diagnoses were categorised into: schizophrenia (including schizophreniform, schizoaffective, delusional disorder and psychotic disorder not otherwise specified), bipolar disorder, major depressive disorder, brief psychotic disorder, and other mental disorders. A psychiatrist assessed outcome at discharge using the clinical global impression (CGI). Co-morbidities such as medical–surgical conditions, substance use disorders, and extrapyramidal side effects were also documented. The use of khat (a commonly abused stimulant substance) was also highlighted. Extra pyramidal side effects included neuroleptic induced Parkinsonism, dystonia, akathisia and tardive dyskinesias.

Data were collected using checklists and questionnaire adopted from previously done research at Amanual Hospital, Addis Ababa to collect the required information for this study purpose. Two psychiatry resident students collected data for 5 days who are familiar with the medical records and that have an experience in data collection. The principal investigators trained data collectors for 1 day about the objective and purpose of the study. The quality of the data collection was monitored by giving clear instructions to data collectors, checking sample filled questionnaire and checklists checked by the principal investigators at daily bases and instance instruction was given on the spot if any incomplete or mistake appeared as well as through double entry of the collected data.

### Data analysis

Data were entered into Epi Data version 3.1 for data exploration and cleaning. The cleaned data were exported into SPSS version V. 20 software for analysis. Descriptive statistics such as measures of central tendency, frequencies, and percentages were computed and presented. Bivariate logistic regression was done to explore associations between variables of interest and patients recovery/outcomes. Predictors of outcomes were assessed using the multivariate logistic regression model to adjust for potential confounding effects with list-wise deletion for missing values. The outcome was included in the logistic model as the dependent variable. Statistical significance was declared at *P* value of <0.05.

### Ethical statement

The Ethical Review Board of College of Health Science, Jimma University granted ethical approval. The data was collected with no personal identifiers at all stage of the study.

## Result

### Background characteristics

The use of abusive psychoactive substances nearly half of the patients 206 (51.2%) were abused psychoactive substances such as “khat” (*Catha edulis*) chewing or alcohol drinking or cigarette smoking or either two of them or all of them.

Concerning the previous admission history of patients, 311 (77. 4%) of them were not previously admitted to the psychiatric treatment facility. Among those 402 study participants’ case and diagnosis history, the diagnosis of admission was bipolar disorder which accounts 104 (25.9%), followed by depression and anxiety 101 (25.1%).

The mean length of health facility admission stay was 34 (SD ± 20.7) days with a minimum 1 day and maximum 152 days after admission to the psychiatry admission ward. Proportionally, 103 (25.6%) of them were within 31–44, followed by 102 (25.4%) of them were stayed within the range of 1–20 days and the remaining, 98 (24.4%) stayed 44 days or more and 98 (24.4%) were stayed 20–31 days (Table 1).

**Table 1:** Background-characteristics of psychiatric admission at Jimma University Training and Teaching Specialized Hospital, southwest Ethiopia, 2015

Variables	Categories	Patients status				Total	
		Currently not improved		Currently improved			
		Frequency	Percent	Frequency	Percent	Frequency	Percent
Sex	Male	63	24.0	200	76.0	263	65.8
	Female	36	26.3	101	73.7	137	34.3
Age group	≤24	42	23.6	136	76.4	178	44.5
	25–34	36	27.5	95	72.5	131	32.8
	≥35	21	23.1	70	76.9	91	22.0
Educational status	Not able to read or write	27	24.8	82	75.2	109	27.1
	Grade 1–8	31	39.2	48	60.8	79	19.7
	Grade 9–12	24	20.5	93	79.5	117	29.1
	Grade 12+	19	19.6	78	80.4	97	24.1
Marital status	Single	68	26.3	191	73.7	259	64.8
	Married	26	23.9	83	76.1	109	27.3
	Widowed, divorced	5	15.6	27	84.4	32	8.0
Occupational status	Unemployed	27	22.5	93	77.5	120	29.9
	Farmers	18	31.0	40	69.0	58	14.4
	Governmental employee	11	20.8	42	79.2	53	13.2
	Students	27	24.5	83	75.5	110	27.4
	House wife and merchants	18	29.5	43	70.5	61	15.2
Religion	Muslim	71	29.1	173	70.9	244	60.7
	Orthodox	17	18.5	75		92	22.9
	Protestant and catholic	13	19.7	53	80.3	66	16.4
substance abused	Abuse substances	53	25.7	153	74.3	206	51.2
	Not abuse substances	48	24.5	148	75.5	196	48.8
Previous admission history	Yes	30	33.0	61	67.0	91	22.6
	No	71	22.8	240	77.2	311	77.4
Types of diagnosis	Schizophrenia	22	22.7	75	77.3	97	24.1
	Depression and anxiety	27	26.7	74	73.3	101	25.1
	Bipolar disorder	28	26.9	76	73.1	104	25.9
	Brief psychotic disorder	24	24.0	76	76.0	100	24.9
Length of admission stay	1–20 days	23	22.5	79	77.5	102	25.4
	21–30 days	26	26.5	72	73.5	98	24.4
	31–44 days	26	25.2	77	74.8	103	25.6
	>44 days	24	24.7	73	75.3	97	24.6

### Predictors of psychiatric patient’s outcomes after admission to health facility

In order to predict the likelihood of the outcomes of the admission of the psychiatry cases, multiple logistic regression was used and the overall model to predict probability of improving patients psychiatric cases in respondents was statistically significant [−2Loglikelyhood = 231.808, X^2^ = 109.749, df = 11 with a P value <0.0001] and the overall prediction of the model was 80.5%.

In multiple logistic regression patients with educational status being at 1–8th-grade level had 1.34 times more likely to improve than those patients who are not able to read or write [AOR = 1.34, 95% CI (1.18–2.78), P < 0.009)]. Nevertheless, those patients in 7–12th grade or above 12th grade had no statistical association with the outcomes. The odds ratio for marital status indicates the probability of client’s psychiatric improvements among married patients were 2.81 times more likely to improve than patients who were single [AOR = 2.81, CI (1.90–4.50), P < 0.043)]. However, those widowed and divorced patients had no statistical association with client’s psychiatric improvement status.

The probability improvements in psychiatric admitted patients, on those patients who were not previously admitted, had 2.82 times more likely improved than patients who had previous admissions history, [AOR = 2.82, CI (2.05–3.17), P < 0.034]. The odds ratio of patients’ length of stay indicated that, the probability of client’s psychiatric improvement on patients who were stayed in the range of 31–44 days in the psychiatric clinic were found 1.88 times more likely than those patients who stayed in the range of 1–20 days, [AOR = 1.88, CI = (1.42–2.65), P < 0.035)]. However, admission stay for more than 44 days did not show any statistical association with the patient’s improvement status (Table 2).

**Table 2:** Independent predictors of patient’s improvement status, at Jimma University Training and Teaching Specialized Hospital, southwest Ethiopia, 2015

Variables	Categories	Patients status		COR (95% CI)	AOR (95% CI)	P value
		Currently not improved	Currently improved			
Sex	Male	63	200	0.88 (0.55–0.42)		
	Female	36	101	1		
Age group	≤24	42	136	1		
	25–34	36	95	0.81 (0.49–1.37)		
	≥35	21	70	1.03 (0.57–1.87)		
Education	Illiterate	27	82	1		
	Grade 1–8	31	48	0.51 (0.27–0.95)	1.34 (1.18–2.78)	0.009
	Grade 9–12	24	93	1.28 (0.68–2.38)		
	Grade 12+	19	78	1.35 (0.70–2.62)		
Marital status	Single	68	191	1		
	Married	26	83	1.14 (0.68–1.91)	2.81 (1.90–4.50)	0.043
	Widowed, divorced	5	27	1.92 (0.71–5.19)		
Occupation	Unemployed	27	93	1		
	Farmers	18	40	0.65 (0.32–1.30)		
	Gov’t employee	11	42	1.11 (0.50–2.44)		
	Students	27	83	0.89 (0.48–1.64)		
	House wife or merchants	18	43	0.69 (0.34–1.39)		
Religion	Muslim	71	173	1		
	Orthodox	17	75	1.81 (1.00–3.28)	1.81 (1.00–3.28)	0.0001
	Protestant or catholic	13	53	1.67 (0.86–3.26)		
substances abused	Abuse substances	53	153	1	1	
	Not abuse substances	48	148	1.07 (0.68–1.68)		
Previous admission	Yes	30	61	1	1	
	No	71	240	1.66 (0.10–2.77)	2.82 (2.05–3.17)	0.034
Patient’s diagnosis	Schizophrenia	22	75	1	1	
	Depression and anxiety	27	74	0.80 (0.42–1.54)		
	Bipolar disorder	28	76	0.80 (0.42–1.52)		
	Brief psychotic disorder	24	76	0.93 (0.48–1.80)		
Length of stay	1–20 days	23	79	1	1	
	21–30 days	26	72	0.81 (0.42–1.54)		
	31–44 days	26	77	0.86 (0.45–1.64)	1.88 (1.42–2.65)	0.035
	>44 days	24	73	0.89 (0.46–1.70)		

## Discussion

There are four main findings from this analysis. First, married patients were 2.81 times more likely to improve than patients who are single. Nevertheless, those widowed and divorced patients were found to have no statistical association with client’s psychiatric improvement status. This might be due to the reason that a husband or wife might play a great role in the psychiatric case management. This study finding is supported by a research done by Ithman MH, Gopalakrishna G, indicating that being unmarried, divorced, or widowed are indices of social disorganisation that had shown significant implications for mental health [4].

Psychiatry cases admitted patients who were the elementary school (1–8th grade) were 1.34 times more likely to improve than those patients who were either illiterate or above 12th-grade. This might be due to the reason that the more the educated; less counselling session will be given by the psychiatric counselors. Those who are not able to read or write might not listen carefully to the counselling session and counsellor. However, previous studies in Ethiopia showed illiterates were at higher risk for mental illness and slow in recovery [9, 11].

The probability of client’s psychiatric improvements in those patients who were not previously admitted is 2.82 times more likely to improve than patients who had previous admissions history. This might be due to the reason that those patients who had been admitted previously might have different category of psychiatric illness or there might be precipitating factors outside of the medical model of patient management and needs careful rigorous study than the current type of study. In line with this substantial body of researches in high-income countries had identified several potential predictors of readmission following a psychiatric illness, hospitalization, poor access to post-discharge, outpatient services, younger age, and more severe clinical diagnosis significant others (being unmarried, divorced, or widowed), medication non-adherence, and poor access to adequate housing [9].

Women’s are less likely than men to be admitted in psychiatric word in our study and the finding is supported by the study carried out to find the pattern of psychiatric illness admitted in psychiatric units in England [12, 13], Bangladesh [14] and at Amanuel Hospital, Addis Ababa, Ethiopia where 3/4th were males [15]. This might be explained by the cultural factor that women do not seek medical attention for their psychiatric problems or it may be due to the reason that the risk of using psychoactive substances among women would be lower than males in our case study.

In the current study, 104 (25.9%) of them were diagnosed bipolar disorder followed by 101 (25.1%) depression and anxiety simultaneously. This finding is similar to other studies, which stated that depression and anxiety were the main recorded diagnosis for psychiatric admission [12, 16].

Patient’s length of stay indicates that the probability of patient’s psychiatric improvement among those who had stayed in the range of 31–44 days in the psychiatric ward were 1.88 times more likely than those patients who stayed in the range of 1–20 days. However, admission stay for more than 44 days does not show any statistical association with the patient’s outcomes. This finding is supported by a systematic review done by Ohnstone P, and Zolese G, indicating that longer hospital stays had not had better mental health care, improved social adjustment or diminished psychopathology [17]. However, one study done in Japan found that strong positive correlation with length of stay [18]. Nevertheless, in a resource poor country, where our study is done, if stayed more in admission ward or clinic lack of the social, emotional and psychological support is more common since the setting is not able to fulfill all these except the medical treatment.

## Limitations of the study

This study is based on a retrospective review of the database in JUTH. Lack of control on the transcriptions in the charts has limited the information we can access to shed a better light on the topic of improvement of the psychiatric case. We encountered incomplete charts and missing data on demographics such as occupation, marital status and education. This lack of information could have altered our results and skewed our results. Our results could not be generalizable to other settings. Finally, causal relationships could not be drawn from our data analysis and our study did not answer whether outcomes associated with higher relapse and frequent re-hospitalization.

## Conclusions and recommendations

Despite the study limitations enumerated above, married patients, elementary school completed patients, previously not admitted and length of stay were strong predictor of outcomes psychiatric patients. Therefore, it is clear that, beyond the medical model of managing the psychiatric patients, psychiatric case management needs the collaborative care of the family in concurrence with definite and focused counselling approach based on the patient’s educational background with enough periods by the counsellors and psychiatrists are central. There should be room to assist families and relatives so that they can be fully involved in the case management and care of the psychiatry admitted cases.
